# Comment on: Probabilities of conditionals: The relevance effect might be confounded by the existence of boundary cases (2025) by Zhan and Wang

**DOI:** 10.3758/s13423-026-02889-5

**Published:** 2026-03-17

**Authors:** Niels Skovgaard-Olsen, Karl Christoph Klauer

**Affiliations:** Department of Psychology, University of Freiburg, Freiburg im Breisgau, Germany

**Keywords:** Conditionals, Probabilities, Statistical error

## Abstract

Zhan and Wang (2025) claim that the relevance effect found in Skovgaard-Olsen et al. (2016) might be an artifact due to “boundary cases” of zero or one probabilities. The relevance effect refers to the finding that people’s expectations about the probabilistic relevance of the antecedent for the consequent influences people’s probability judgments of indicative conditions, “If A, then C”. In Skovgaard-Olsen et al. (2016, 2017), this effect was found not by using probabilistic notions of relevance, like ΔP=P(C|A)-P(C|-A), as direct predictors of P(if A, then C). Instead, stimulus materials were pre-validated to implement different qualitative reason relation assessments of whether A is a reason for C, A reason against C, or neither, which were operationalized via categories of probabilistic relevance (positive relevance ΔP>0, negative relevance ΔP<0, irrelevance ΔP=0). Despite this, this commentary addresses the fact that the binomial statistical model used by Zhan and Wang (2025) is invalid for the type of data collected. The purpose of this paper is to do a re-analysis of the two data sets of Zhan and Wang (2025). First, we reanalyze the new data that Zhan and Wang (2025) present. Then we re-analyze the data from Skovgaard-Olsen et al. (2016), which Zhan and Wang (2025) in turn re-analyze in Table 5 of their paper. In both cases, we find that invalid statistical models were applied and that the conclusions that Zhan and Wang (2025) draw must be rejected once valid statistical models are applied.

Zhan and Wang (2025) claim that the relevance effect found in Skovgaard-Olsen et al. (2016) might be an artifact due to “boundary cases” of zero or one probabilities. The *relevance effect* refers to the finding that people’s expectations about the probabilistic relevance of the antecedent for the consequent influence people’s probability judgments of indicative conditions, “If A, then C.” In Skovgaard-Olsen et al. (2016, 2017), this effect was found not by using probabilistic notions of relevance, like $\Delta P=P\left(C|A\right)-P(C|\overline{A})$, as a direct predictor of P(if A, then C). Instead, stimulus materials were pre-validated to implement different qualitative reason-relation assessments of whether A is a reason *for* C, a reason *against* C, or *neither*, which were operationalized via categories of probabilistic relevance (positive relevance $\Delta P>0$, negative relevance $\Delta P<0$, irrelevance $\Delta P=0$). In contrast, Zhan and Wang (2025) use probabilistic notions of relevance such as $\Delta P$ as a direct predictor of P(if A, then C) and test for its interactions with P(C|A), despite the fact that (1) $\Delta P$ takes values in [-1,1] and P(if A, then C) is constrained to [0,1]; so $\Delta P$ is not a suitable replacement for P(C|A) in P(if A, then C) = P(C|A), and (2) relevance indices like $\Delta P$ are defined in terms of P(C|A). In Skovgaard-Olsen et al. (2016) such an approach was considered and rejected on *a priori* grounds. Similar points hold for other relevance operationalizations that Zhan and Wang (2025) consider.^1^ Moreover, we do not see any substantial reasons for filtering out P(C|A) = 1 cases in evaluating the influence of relevance on P(if A, then C), since proponents of a relevance approach to conditionals will argue that indicative conditionals can be defective despite P(C|A) = 1, if the antecedents are irrelevant for the consequents (Skovgaard-Olsen, 2016). Despite this, this commentary focuses on the fact that the statistical model used by Zhan and Wang (2025) is invalid for the type of data collected.

We did a re-analysis of both Zhan and Wang’s (2025) new data and their reanalysis of the data from Skovgaard-Olsen et al. (2016), and discovered some fundamental errors, which invalidate their analysis and conclusions. The basic error is to model transformed continuous ratings of participants (ratings on a scale from 0% to 100% transformed to [0,1] by dividing by 100) as Bernoulli trials via a binomial likelihood with number of trials set to 1. The binomial likelihood function expects counts of successes among a fixed number of trials. However, Zhan and Wang (2025) use this model, without providing a rationale in the paper, for modelling continuous variables in [0,1] or (0,1), when they conduct analyses that contrast the full data sets with subsets of the data created by filtering out ‘0’ and ‘1’ values. Surprisingly, although only the values ‘0’ and ‘1’ are admissible as data values in their analysis approach, the software used by Zhan and Wang (2025) does not crash under conditions, producing *p* values. These values are, however, devoid of meaning and in particular cannot be interpreted with reference to statistical significance. With slight changes to their code, the software will crash, however, when attempting to compute the log-likelihood of their invalid model, as we show in a Jupyter notebook on the Open Science Framework (OSF).

In reply to this argument, one reviewer defended the analysis of Zhan and Wang (2025) but replaced wts = 1 with wts = 100 in the Binomial likelihood function, effectively treating each 0–100% rating as if it were the outcome of 100 independent Bernoulli trials. While this adjustment allows their model to converge, it does not resolve the fundamental mis-specification. Subjective probability ratings are not counts of successes in repeated Bernoulli trials but bounded continuous judgments. Interpreting, for example, a 49% rating as “49 successes out of 100 trials” is a category error. QQ-plots of the fitted model reveal the mis-specification.

In their models, Zhan and Wang (2025) moreover include image-wise random slopes for predictors. However, given their experimental design, these predictors were manipulated to be constant within each image, and so there is not enough information to estimate image-specific random slopes and their interaction.

Zhan and Wang (2025) formulate their hypothesis that boundary ‘0’ and ‘1’ cases influence the relevance effect for a data set that contains high proportions of these extreme cases (and NaN values). Their argument relies on comparing models fitted to data sets cleaned of missing values with models fitted to a subset of the full data in which the analysis is restricted to cases where all four contingency cells were non-zero. This reduced dataset only accounts for around 31% of the original observations. However, no power analysis is reported for whether significant interaction effects could be found for this subset of the sample.

Instead of cherry picking among the observations in this way, a more statistically sound approach would be to fit a mixture model to the full data set with appropriate likelihood functions. For instance, models with a zero-one-inflated beta likelihood capture the behavior of participants with extreme 0 and 1 cases with Bernoulli likelihood mixture components while modelling the remaining (0,1) interval by a beta-likelihood and investigating whether credible effects of relevance measures occur for the remaining interval. This approach captures the observed inflation at the extremes while retaining statistical power from the full dataset. When this model is fitted, significant interaction effects emerge for several of the relevance indices.

In their Table 5 when re-analyzing the data from Skovgaard-Olsen et al. (2016), it is only when Zhan and Wang (2025) use their invalid binomial model fitted to their reduced data set that they fail to find an interaction with the relevance factor. With the linear mixed-effects model such an interaction is found, even for the reduced subset, and applying a logit transformation shows that the effect is robust under a nonlinear transformation that maps the interval (0,1) onto the real line, even for the reduced subset.

Zhan and Wang (2025) claim that the relevance effect is not found for extensional probabilities. However, Skovgaard-Olsen et al. (2021, Experiment 2) replicated the relevance effect using a trial-by-trial learning format in which participants were provided with events explicitly through animations, instead of having to mentally simulate these based on background knowledge from working memory. Furthermore, Zhan and Wang (2025) mis-characterize the relevance effect when they claim that it depends on the requirement of an inferential relation between the antecedent and consequent for a conditional to be accepted as “true” (p. 2). In contrast, the relevance effect reported in Skovgaard-Olsen et al. (2016) concerns the probability and acceptability of conditionals, and in Skovgaard-Olsen et al. (2017) it is found to not generalize to truth value assignments.

We have uploaded this re-analysis on the OSF as a Jupyter notebook but here repeat the main conclusions:^2^

1. Zhan and Wang’s (2025) analysis uses models with a binomial likelihood function which do not match the data-generating process. Such binomial models are incorrect for the continuous data measured; hence, the *p* values that the authors report to substantiate their hypothesis for both data sets are invalid.
2. The Julia code used by the authors does not immediately reveal that the binomial model is invalid. This is because the model calls programmed by the authors produce estimates and *p* values without warnings or error message. However, when the same model is fitted interactively or evaluated in full, an error *is* issued indicating that the data format of the outcome variable is incompatible with the binomial likelihood.
3. Setting the weight of binomial models to 100 instead of 1 (as suggested by one reviewer) does not solve the problem. The QQ-plots show pathological behavior and the models remain conceptually inadequate.
4. The authors use models that include image-wise random slopes: (1 + CP * DP|image). However, given the experimental design of Zhan and Wang (2025), CP and DP were manipulated to be constant within each image and there is not enough information to estimate separate effects for an intercept, CP, DP, CP×DP within each image. Only an image-specific random intercept can be estimated.
5. The authors’ analysis rests on a subset analysis which only retains 31% of the data.
6. When applying the original mixed-effect model from Skovgaard-Olsen et al. (2016), and comparing it to a reduced dataset where ‘0’ and ‘1’ have been filtered out, the interactions with relevance remain significant.
7. Re-analyzing the data set from Skovgaard-Olsen et al. (2016) using a model that first logit-transforms the outcome variable and then fits a Gaussian mixed model, shows:
  - A significant interaction with relevance is found.
  - Even after filtering out ‘0’ and ‘1’ values, a significant interaction with relevance remains.
8. Re-analyzing the authors’ new data set using zero/one inflated beta-regression models on the full data sets (after removing missing values and infinite values) shows: When controlling for distinct behavior with extreme 0 and 1 values via a Bernoulli mixture component, significant interactions with relevance remain for the beta mixture component handling the open interval (0,1).

Our re-analysis thus demonstrates that once ratings are modeled appropriately as bounded continuous data, significant interactions with relevance remain, undermining Zhan and Wang’s central claim that the effect disappears under proper controls. But since the relevance indices such as $\Delta P$ are defined in terms of P(C|A), and take, for example, negative values outside the [0,1] range (and thus do not represent the probability of “If A, then C”), the analytic choice of Zhan and Wang (2025) of testing for relevance effects by interactions between the relevance indices and P(C|A) was rejected *a priori* in Skovgaard-Olsen et al. (2016) for good reason.

## Data Availability

The data associated with this study are available at https://osf.io/c4xky.

## Footnotes

1 While their fourth relevance measure is scaled to [0,1], it was found to fail empirically in Skovgaard-Olsen et al. (2021).

2 https://osf.io/c4xky

## Code availability

The analysis code associated with this study is available at https://osf.io/c4xky.

## References

[CR1] Skovgaard-Olsen, N. (2016). Motivating the relevance approach to conditionals. *Mind & Language,**31*(5), 555–79.

[CR2] Skovgaard-Olsen, N., Singmann, H., & Klauer, K. C. (2016). The relevance effect and conditionals. *Cognition,**150*, 26–36. 26848733 10.1016/j.cognition.2015.12.017

[CR3] Skovgaard-Olsen, N., Kellen, D., Krahl, H., & Klauer, K. C. (2017). Relevance differently affects the truth, acceptability, and probability evaluations of ‘and’, ‘but’, ‘therefore’, and ‘if then.’ *Thinking and Reasoning,**23*(4), 449–482.

[CR4] Skovgaard-Olsen, N., Stephan, S., & Waldmann, M. (2021). Conditionals and the hierarchy of causal queries. *Journal of Experimental Psychology: General,**150*(12), 2472–2505. 34881947 10.1037/xge0001062

[CR5] Zhan, L. and Wang, M. (2025). Probabilities of conditionals: The relevance effect might be confounded by the existence of boundary cases. *Psychometric Bulletin & Review*. 10.3758/s13423-025-02725-2 10.3758/s13423-025-02725-2 40624400

